# Colorectal cancer in children: an evaluation of the existing literature based on the 11-year experience of a single center

**DOI:** 10.1007/s00383-025-06020-y

**Published:** 2025-04-15

**Authors:** Seyithan Ozaydin, Sumeyra Dogan, Ipek Yildiz Ozaydin, Ali Aycicek, Rabia Ata, Zahit Mahmut, Unal Guvenc, Cemile Besik, Oyhan Demirali

**Affiliations:** 1 Department of Pediatric Surgery, Kanuni Sultan Suleyman Training and Research Hospital/Health Sciences University, Istanbul, Turkey; 2 Department of Pediatric Radiology, Kanuni Sultan Suleyman Training and Research Hospital/Health Sciences University, Istanbul, Turkey; 3 Department of Pathology, Kanuni Sultan Suleyman Training and Research Hospital/Health Sciences University, Istanbul, Turkey; 4 Department of Pediatric Oncology, Kanuni Sultan Suleyman Training and Research Hospital/Health Sciences University, Istanbul, Turkey

**Keywords:** Pediatric colorectal cancer, Mucinous adenocarcinoma, Signet cell adenocarcinoma

## Abstract

**Purpose:**

Children's risk of developing colorectal cancer (CRC) is relatively low. In this report, we present our experience with CRC in the pediatric age group, together with an extensive review of the literature.

**Methods:**

Between the years 2013 and 2024, a total of five patients diagnosed with CRC underwent treatment at the Department of Pediatric Surgery in our tertiary hospital. A retrospective evaluation was conducted on patients' charts, encompassing demographics, admission symptoms, patient and family histories, laboratory and radiologic findings, operative and pathology reports, genetic and molecular study results, treatment protocols, and follow-up data.

**Results:**

There were three males and two females, with a mean age of 13.5 ± 2.5 years. The primary sites were the sigmoid and rectosigmoid. Two patients were diagnosed with mucinous adenocarcinoma based on histopathological examination. Among the three remaining patients, one presented with signet ring cells, one displayed moderately differentiated adenocarcinoma characteristics, and one exhibited well-differentiated adenocarcinoma characteristics. At the time of the most recent follow-up, two patients have demonstrated survival.

**Conclusion:**

The two main factors contributing to poor survival in pediatric CRC were concluded to be at an advanced stage during diagnosis and having an aggressive histologic subtype. Including CRC in the preliminary diagnosis list is essential for an early diagnosis in the pediatric age group.

## Introduction

Colorectal cancer (CRC) is a malignancy known for its unfavorable prognosis and infrequent occurrence in the pediatric population. Diagnosing this condition in children is often delayed due to nonspecific symptoms [1–4]. When considering adults, it is worth noting that it is the third most prevalent malignancy, surpassing all others except for lung and breast cancers. Furthermore, its prognosis slightly improves compared to the pediatric age group [3, 5]. The prevalence of CRC among adults ranges from 0.1% to 6% [6–8], whereas in children, it is much lower at 0.2 to 2 cases per million [2, 9–11]. Notably, the prevalence rate is 0.12 per million in the 0–14 age group, whereas it surges to 1.78 per million in the 15–19 age group, with a subsequent increase observed after age fifteen [12].

Moreover, it is important to highlight that despite the youngest case recorded in the literature being nine months old, the highest incidence occurs at fifteen in children [10]. According to reports, the age at which CRC reaches its highest incidence in adults is sixty-five years [10]. Furthermore, in the case of adults, CRC is predominantly found in males, with a male-to-female ratio of 5.1 to 3.8, as reported in previous studies [8]. Conversely, in children, the incidence of CRC is evenly distributed between males and females, with a male-to-female ratio of 1 to 1, as indicated by multiple sources [1, 10, 12].

Physicians involved in pediatric patients'care should possess knowledge of this rare tumor's presentation and imaging characteristics to facilitate prompt diagnosis and treatment. The purpose of this study was to enhance clinicians'understanding of this disease and streamline the management of pediatric CRC.

## Methods

Following the approval from the Ethics Committee of XXX Hospital (#2023.10.146), a retrospective analysis was undertaken on the medical records of patients diagnosed with CRC in our clinic between 2013 and 2024. The evaluation encompassed demographic data, admission complaints, medical and family history, laboratory and radiologic examinations, operative findings, pathology reports, genetic and molecular study results, chemotherapy, radiotherapy, and patient follow-up processes.

### Documentation of Cases

#### Case 1

**Fig. 1:**
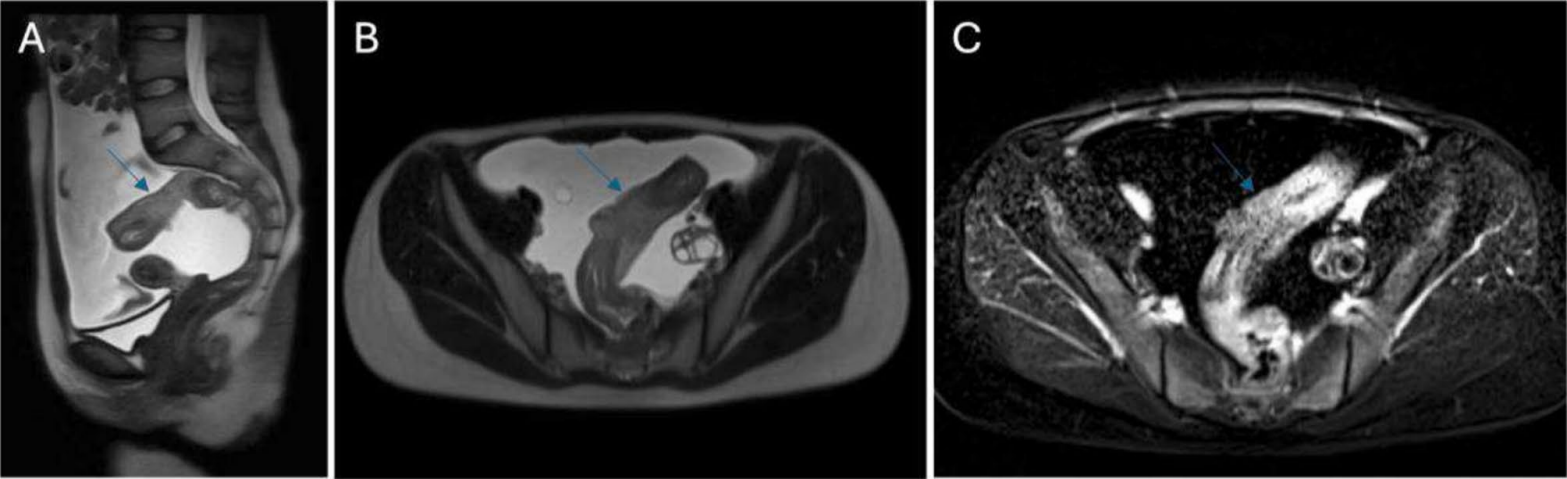
T2 A sagittal (**A**), T2 A axial (**B**), and postcontrast T1 A (**C**) series, demonstrating diffuse wall thickening and contrast enhancement of a long segmental mass in the sigmoid colon with accompanying ascites in Case 1

A female patient, aged sixteen, was admitted to our hospital as a result of intermittent abdominal pain persisting for three months, with a recent escalation in symptoms over the last two days. The sigmoid colon was found to have a mass measuring 12 × 10 × 8 cm, as indicated by ultrasound (US), computed tomography (CT), and magnetic resonance imaging (MRI) (Fig. 1). Additionally, diffuse free fluid was observed intra-abdominally, along with a cystic mass measuring 3 × 4 cm in the left ovary. Tumor markers, carbohydrate antigen 19–9 (CA 19.9) and carbohydrate antigen 125 (CA 125), were slightly elevated. There were no notable findings in her medical and family histories. Surgical intervention was scheduled, and upon exploration, diffuse free fluid was observed within the abdominal cavity. A cytologic sample was collected from this fluid. A mass was detected approximately 5 cm proximal to the peritoneal reflection. It extended about 20 cm proximally within the intestinal segment, completely occupying the colon and protruding beyond the serosa. Adhesions to the surrounding tissues were observed. There were also small white lesions, which exhibited diffuse involvement of the peritoneum and omentum, indicating peritoneal carcinomatosis. Surgical resection was conducted at the location of the mass in the intestine, involving the removal of a 5 cm segment of healthy tissue from both ends. The distal end was closed by creating a Hartmann pouch, and the proximal end was diverted through a colostomy. Lymph node dissection, omentectomy, and appendectomy were performed. A cystic lesion measuring 3 × 4 cm was identified in the left ovary and subsequently removed, while the right ovary, uterus, and vaginal wall presented no anomalies.

**Fig. 2:**
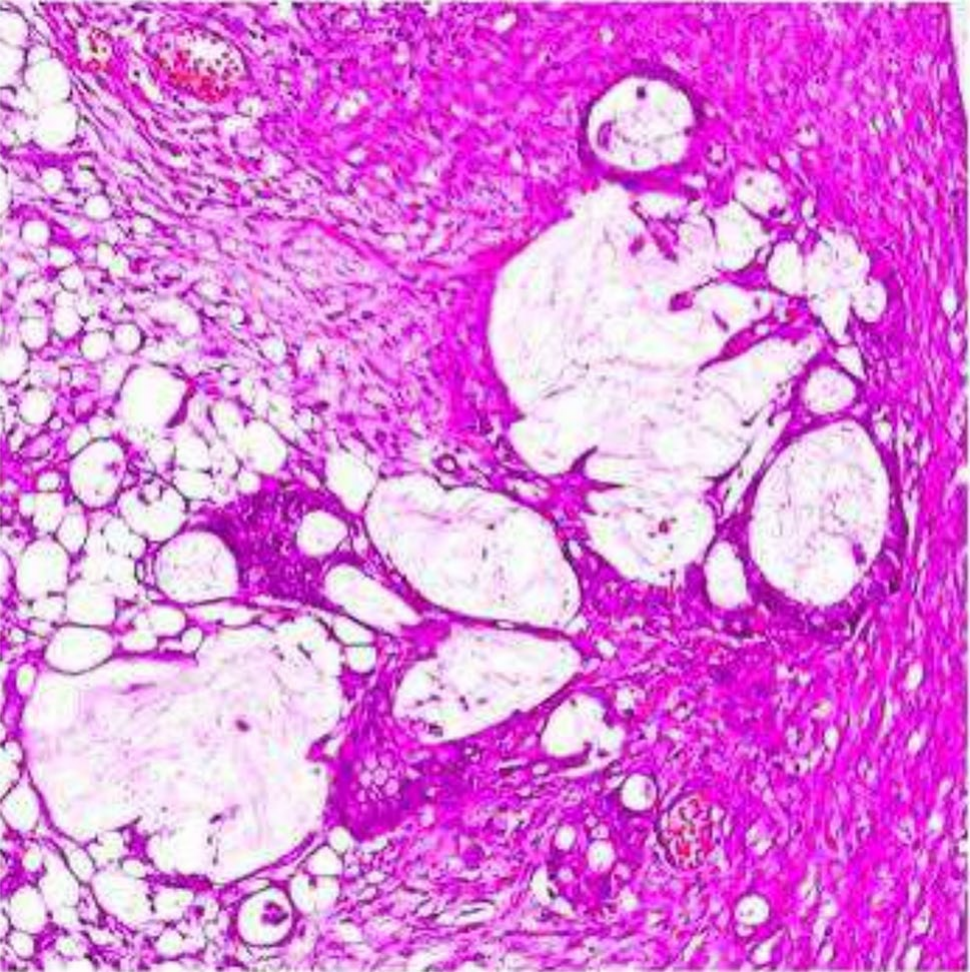
Microscopic pathologic appearance of mucinous adenocarcinoma in Case 1

The pathology report indicated the presence of mucinous adenocarcinoma, with a tumor size measuring 11.2 × 9.8x7.6 cm, which included signet ring cell areas (Fig. 2). The surgical margins were clear. The presence of lymph node involvement was observed, and the cytologic fluid confirmed the presence of tumor cells in the omental and peritoneal lesions. The appendix displayed normal characteristics, and the left ovarian cystic lesion was identified as a corpus luteum cyst. The genetic and molecular findings yielded normal results. No expression losses were detected for Human mutL homolog 1 (MLH-1), Human postmeiotic segregation increased 2 (PMS-2), Human mutS homolog 2 (MSH-2), and Human mutS homolog 6 (MSH-6). After transferring to the Oncology department, the patient underwent evaluation, resulting in a Stage 3 C diagnosis, followed by the commencement of chemotherapy using the Folfox protocol (5-Fluorouracil, Folinic acid, and Oxaliplatin), and subsequently, radiotherapy was initiated. After two months, a surgical operation was performed on her due to the recurrence of ileus.

During the exploration, diffuse free fluid and dense adhesions were observed. A frozen pelvis had developed due to the conglomeration of intestinal segments. While freeing the adhesions, serosal defects occurred, especially up to 30 cm proximal to the ileocecal valve. A resection was performed on both colon and terminal ileum. Subsequently, an ileostomy was created. The pathology report indicated the presence of tumor cells in both colon and cytologic fluid. While the Oncology department continued her treatment, she developed ileus again three months later. She underwent exploration due to the malfunction of the ileostomy. Adhesions were freed, and the ileostomy was disrupted. An additional 100 cm of small intestine was surgically removed, and a proximal ileostomy was subsequently performed. As part of the exploration, biopsies were performed on the diffuse metastatic lesions present on the surface of the liver. Once again, the findings were consistent with mucinous adenocarcinoma featuring signet ring cell components. Carcinoembryonic antigen (CEA) and CA 19.9 levels were found to be elevated during this period. The patient died two months later.

#### Case 2

**Fig. 3:**
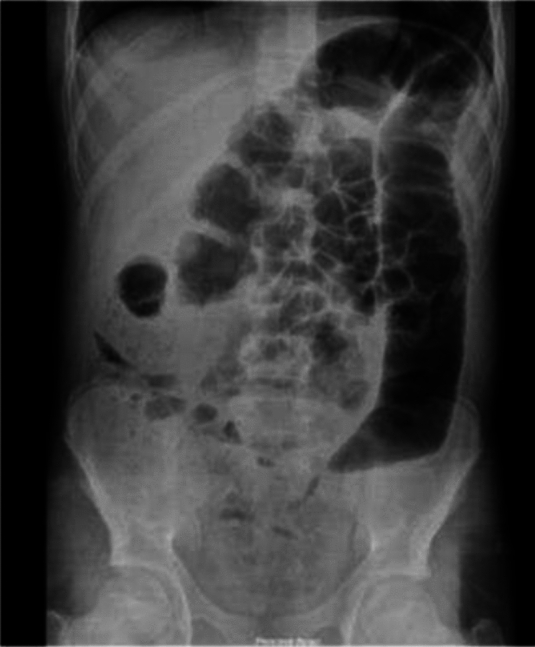
The appearance of a cut-off sign in the rectosigmoid, indicating an obstruction of the distal passage and proximal gas distension in Case 2

A 15-year-old male patient presented to the hospital with symptoms of abdominal pain and distention. The past medical history showed no unusual findings. Upon examination, diffuse abdominal tenderness was noted, accompanied by gas distention. No discernible mass was detected, and rectal examination produced normal findings. As per the imaging findings, a 4 × 3x2 cm mass was identified in the sigmoid colon, leading to partial obstruction (Fig. 3). The tumor markers demonstrated normal results.

A minimal quantity of free fluid was observed in the abdominal cavity during laparotomy, and a cytologic specimen was obtained. The surgical exploration revealed that a mass in the sigmoid colon had extended to the serosa, located 25 cm proximal to the peritoneal reflection. A nodular lesion measuring 2 × 2 cm was detected in the omentum. Surgical excision of the colon mass was performed, involving the removal of a 5 cm segment of normal bowel from both ends, followed by the creation of a colo-colic anastomosis. Lymph node dissection, omentectomy, and appendectomy were performed.

The pathology report indicated the presence of mucinous adenocarcinoma, with a tumor diameter measuring 3.2x.2 × 1.4 cm. The appendix was normal. The surgical margins were clear, and lymph node involvement and tumor cells in the cytologic fluid were present. It was noted that there was adenocarcinoma infiltration in the omental lesion. The genetic and molecular assessments revealed no abnormalities. No indications of expression losses were found for MLH-1, PMS-2, MSH-2, and MSH-6.

The commencement of chemotherapy, employing the Folfox protocol, was implemented under the purview of the oncology department for stage 3 C, followed by radiotherapy. No pathologic findings were observed through radiologic or endoscopic examination until the sixth month of follow-up. In month ten, a mass measuring 3 cm was found in the rectosigmoid region, accompanied by a 28 mm metastatic nodule in the liver. The combination of positron emission tomography (PET) and CT demonstrated elevated tracer uptake in liver segments 2, 4, 6, and 8, as well as in the mesentery of the small intestine, the peritoneal and rectal colon wall, and numerous metastatic lymph nodes in the paraaortic and pararectal regions. The radiologic findings indicated the presence of grade 2 hydronephrosis in the right kidney and the complete absence of the distal right ureter. Subsequently, the patient was transferred to the operating room, where a meticulous exploration uncovered the presence of significant and tightly bound adhesions. A frozen pelvis was observed.

Adhesions were freed. The colon was surgically excised in its entirety, resulting in the establishment of an ileostomy. The lymph node excision procedure was performed. Pronounced adhesions, particularly as a response to radiotherapy, were found to cause significant obstruction in the distal section of the right ureter and, consequently, hydronephrosis. A loop ureterostomy was performed on the middle portion of the ureter. Once again, the pathology results confirmed the presence of mucinous adenocarcinoma. Liver nodule and lymph nodes were identified as sites of metastasis. The chemotherapy regimen was supplemented with methotrexate and capecitabine. After a three-month interval, the patient underwent re-exploration due to the occurrence of severe ileus and the failure of the ileostomy. The procedures of adhesiolysis, ileal resection, and proximal ileostomy were executed. However, the patient died one month later. Elevated values of CEA and CA 19.9 were observed during this recurrence period.

#### Case 3

**Fig. 4:**
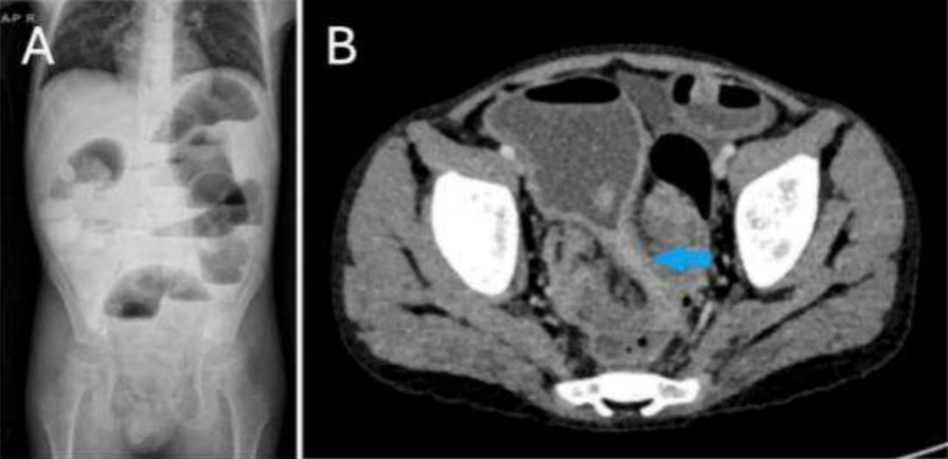
The erect direct abdominal radiograph (**A**) exhibiting diffuse air-fluid leveling within the colonic loops, indicating ileus. The same patient's axial contrast-enhanced CT scan (**B**) revealed substantial rectosigmoid wall thickening, resulting in lumen narrowing (arrowed area) and colonic loop dilation proximally in Case 3

**Fig. 5:**
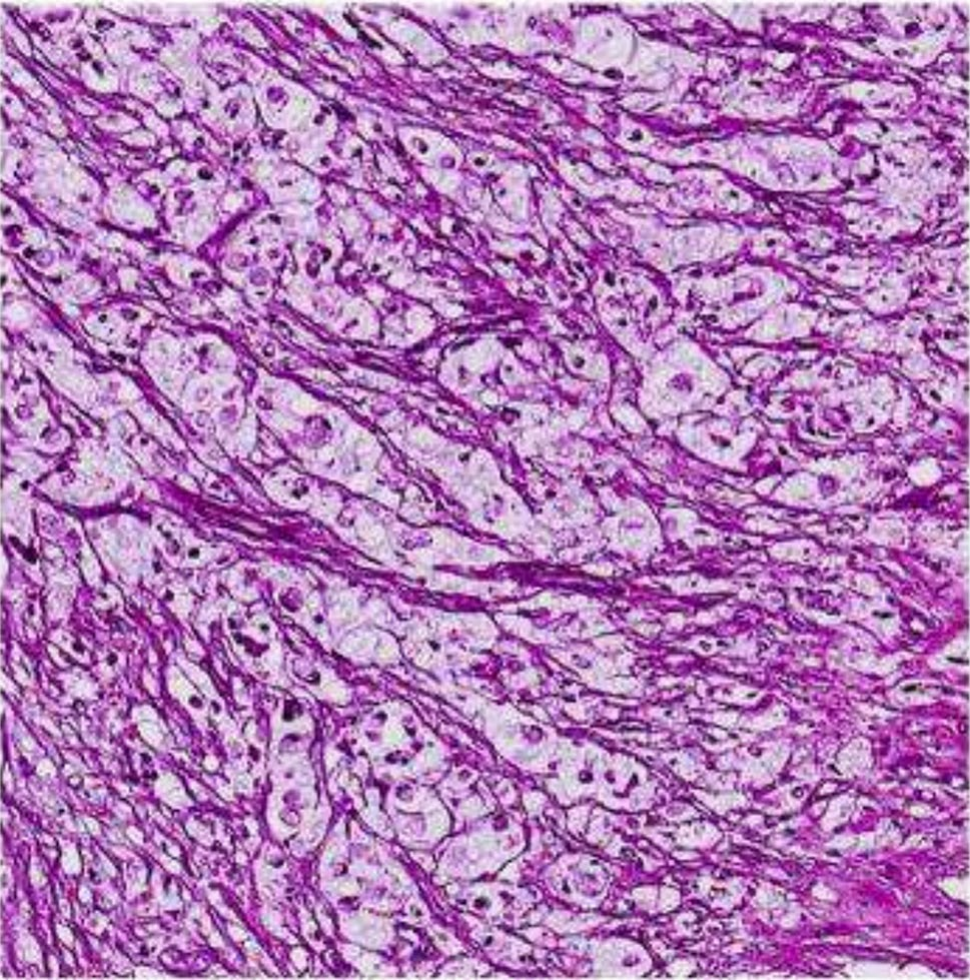
Microscopic pathologic appearance of signet-cell adenocarcinoma in Case 3

An 11-year-old male patient was referred to an external medical facility due to a primary complaint of abdominal discomfort and the presence of blood in stools, which had persisted for 2–3 months. The patient had been diagnosed with B-ALL at the age of three and had undergone successful treatment at the age of five. A review of the patient's family history revealed that both grandfathers had undergone a prior colectomy for colon cancer. A CT scan revealed the presence of a mass measuring 7.5 × 5 × 4 cm in the rectosigmoid region (Fig. 4). The tumor markers exhibited values within the acceptable range. A colonoscopy was performed, and the biopsy findings revealed the presence of signet ring cell adenocarcinoma. The patient underwent a complex surgical procedure, which involved a total colectomy with ileo-anal anastomosis, omentectomy, appendectomy, and lymph node dissection. Unfortunately, anastomotic leakage occurred shortly after the procedure, necessitating re-exploration of the ileostomy. The pathology report indicated the recurrence of signet ring cell adenocarcinoma (Fig. 5). The dimensions of the tumor were recorded as 7.6 × 5.2 × 3.4 cm, and no tumor cells were identified in the surgical margins. Evidence of lymph node involvement was observed, while the omentum and the appendix appeared normal. The patient was subsequently referred to our institution for further evaluation and medical care. The Oncology Department initiated a Folfox protocol chemotherapy regimen in the context of Stage 3 C, which was subsequently followed by radiotherapy.

Nevertheless, the patient was required to undergo surgical intervention on two occasions due to the onset of ileus. Following a comprehensive examination, it was evident that the abdomen exhibited tightly bound intra-abdominal adhesions and the presence of diffuse tumor foci. The lesions were subsequently removed via surgical means, the existing ileostomy was disrupted, and a segment of approximately 100 cm of small bowel was resected, followed by the construction of a more proximally located ileostomy. The pathology report indicated diffuse infiltration in all tissues, including the intestines. The results of the genetic and molecular analyses were within normal limits. The chemotherapy regimen was augmented with the inclusion of methotrexate and bevacizumab, yet the patient died after four months. Elevated levels of CEA and CA 19.9 were detected during the relapse.

#### Case 4

A 15-year-old female patient had undergone a laparoscopic appendectomy at an external medical facility due to an acute abdomen. However, she was subsequently referred to a gastroenterologist due to the persistence of abdominal discomfort. CT and MRI revealed a wall thickness of 9–13 mm in a 5–6 cm segment of the rectosigmoid. A colonoscopy was performed, which revealed wall thickening in the rectosigmoid, resulting in partial narrowing of the passage. The biopsy result was reported as moderately differentiated adenocarcinoma. A neoplastic lesion had not been identified in the surgical specimen obtained during the initial appendectomy.

**Fig. 6:**
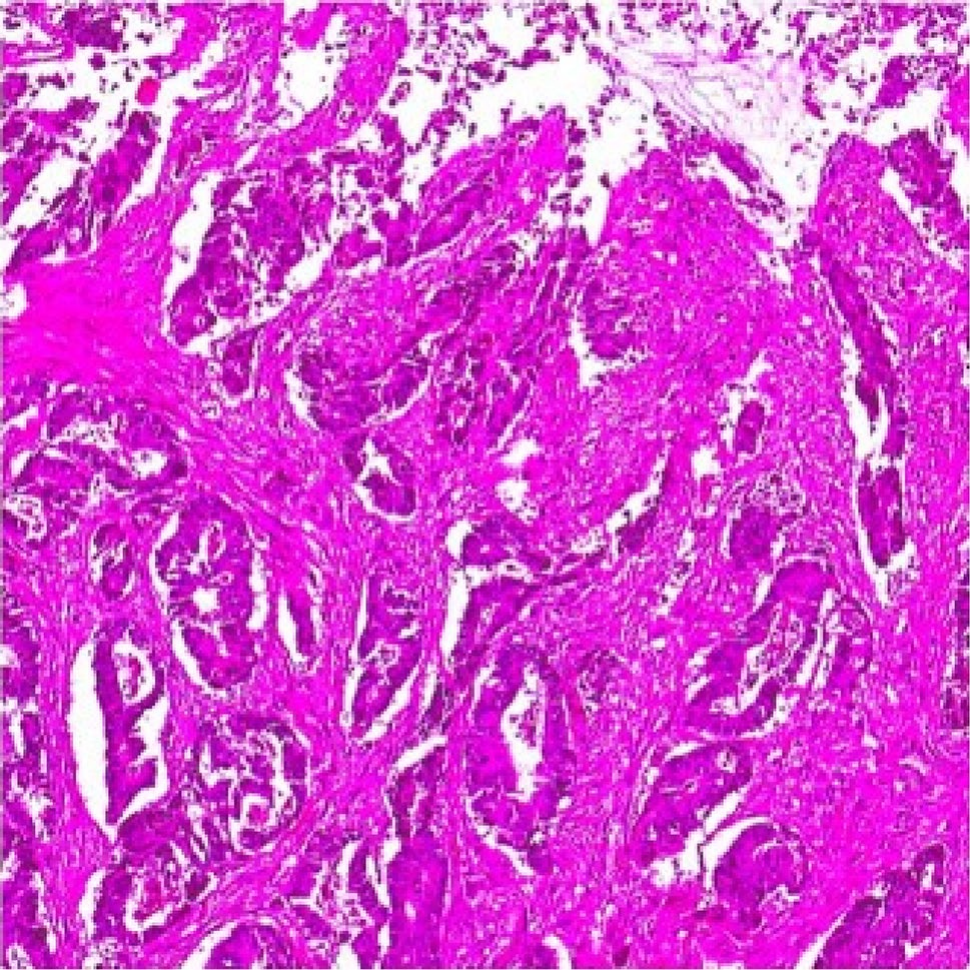
Microscopic pathologic appearance of moderately differentiated adenocarcinoma in Case 4

The patient's medical history revealed no notable abnormalities. The tumor markers were within the normal range. The patient was referred to our hospital, admitted to our clinic, and subsequently investigated. There was minimal intra-abdominal free fluid, and a cytologic sample was obtained. The colonic mass, located 10 cm proximal to the peritoneal reflection, did not exceed the serosa and was identified through palpation. It was subsequently resected with 5 cm normal bowel segments from both ends, followed by colo-colic anastomosis. A lymph node dissection and omentectomy were also conducted. The pathology report indicated the presence of moderately differentiated adenocarcinoma (Fig. 6). The tumor invaded the muscular layer, with a diameter of 2.5 × 1.5 × 1.6 cm. The surgical margins were free of malignancy, the omentum was normal, there was no evidence of lymph node involvement, and the cytologic examination yielded unremarkable results. The genetic and molecular findings fell within the expected range. The patient underwent chemotherapy according to the Folfox protocol, as prescribed by the Oncology Department, due to the Stage 3 condition. Subsequently, the ovaries were laparoscopically relocated from the pelvis to the anterior abdominal wall, and radiotherapy was administered to the pelvic region. The patient is in the tenth year of follow-up and remains without complications. Regular blood tests, radiologic assessments, and endoscopic examinations are conducted annually for ongoing monitoring.

#### Case 5

A 15-year-old male patient presented with a three-month history of intermittent abdominal pain, diarrhea, vomiting, fever, bloody stools, and weight loss. The patient's medical history was devoid of any significant findings. Nonetheless, his familial background disclosed a prevalence of gastric cancer in his paternal grandmother and colon cancer in his grandfather. The tumor markers exhibited values that were within the normal range. MRI findings demonstrated the presence of a 10 × 4x3 cm mass in the sigmoid colon, along with hyperechogenicity of the surrounding mesenteric fatty tissue and multiple millimetric-sized lymph nodes. There was also a heterogeneous mass extending from the anterior aspect of the intestinal loop to the left transverse abdominal muscle, which might have indicated an enterocutaneous fistula, and was reported as"Although this is not a typical presentation, it is suggestive of a CRC that had developed on an inflammatory background."The levels of all tumor markers fell within the normal range. During the exploratory laparotomy, a minimal quantity of free intra-abdominal fluid was found, prompting the need for a cytologic sample. It was concluded that the structure originally identified as a fistula tract did not conform to the definition of a fistula tract; rather, the mass located in the sigmoid region was observed to be protruding toward the left inguinal canal and was readily dislodged from the inguinal canal. Consequently, the mass situated in the sigmoid region was effectively excised via resection-anastomosis, incorporating 5 cm-long unaffected segments of the bowel at both terminations. Lymph node dissection, omentectomy, and appendectomy were performed.

The pathology report revealed a diagnosis of well-differentiated adenocarcinoma. The tumor exhibited muscularis propria invasion and measured 9 × 3x2.5 cm in diameter. The surgical margins exhibited no signs of tumor, while both the omentum and appendix appeared normal. No lymph node involvement was detected, and the cytology results were negative. Genetic and molecular studies revealed KRAS (+), BRAF (−), normal expressions of MLH-1 and PMS-2, and loss of nuclear expression in MSH-2 and MSH-6. The analysis of microsatellite instability (MSI) led to the identification of Lynch syndrome. The Folfox protocol chemotherapy regimen was initiated in the context of Stage 3 for the patient, who then underwent radiotherapy. In the course of the patient's fourth year of follow-up, the annual examinations have transpired without any remarkable incidents, signifying normal results for blood tests, radiologic evaluations, and endoscopic findings.

### Synopsis of the Cases

Of the five cases, three were boys (60%) and two were girls (40%). The mean age was 13.5 ± 2.5 years. Three patients were admitted to the hospital with complaints of abdominal pain, diarrhea, bloody stools, and weight loss, while two patients presented with ileus. One patient had a history of treatment for B-ALL, while none of them had a history of polyposis syndrome or inflammatory bowel disease. Two patients had a family history of gastric and colon cancer. US, MRI, and CT examinations were performed in all of our patients, and colonoscopy was performed in two patients. Without exception, the mass was situated in the rectosigmoid colon. The mean tumor diameter was 6.7 cm, ranging from 2.5 to 11.2 cm.

One patient received surgical treatment at an external facility. In one case, the distal end of the intestine was closed, while the proximal end was converted into a stoma using Hartmann's closure technique post-resection. During the early stage, three cases with a histopathologic diagnosis indicating a poor prognosis (mucinous and signet ring cells) were lost, while two cases with favorable histology (well-moderately differentiated) and uneventful clinical course have continued to be monitored. According to the results of genetic and molecular studies, such as MLH-1, PMS-2, MSH-2, and MSH-6, only one case exhibited a loss of nuclear expressions in MSH-2 and MSH-6. The diagnosis of Lynch syndrome (LS) was confirmed based on the MSI results. Our LS case was the only one that exhibited KRAS positivity.

The pathology specimens of all cases showed clear surgical margins, and resection was conducted with a minimum of 5 cm of normal bowel segment on either side of the mass location. An external center carried out a similar surgical procedure in a single case. Additionally, all cases underwent cytologic sampling, followed by appendectomy, omentectomy, and lymph node dissection. As per the TNM classification, all patients within our study were diagnosed with stage 3. In light of this, they were all administered a combined therapeutic protocol comprising chemotherapy and radiotherapy. The Folfox protocol was supplemented with various chemotherapeutic agents (Methotrexate, Capecitabine, Bevacizumab) in three cases exhibiting a poor prognosis. Three cases with poor histology exhibited recurrences, leading to the need for multiple surgical interventions as a result of ileus. Elevated CEA and CA 19.9 levels were observed in all three recurrent cases throughout the follow-up period. The data of the cases are summarized in Table 1.

**Table 1:** Demographic, clinical, and histopathological characteristics of our pediatric colorectal cancer cases

Case #	Gender Age (years)	Symptoms	Imaging result	Initial surgical procedure	Histology	Chemotherapy/radiotherapy	Prognosis
1	F, 16	Intermittent abdominal pain (3 months)	Sigmoid mass (12 × 10 × 8 cm)	Resection of the mass and Hartmann's procedure	Mucinous adenocarcinoma	FOLFOX protocol (Stage 3 C)/Radiotherapy	Died 7 months after the first operation
2	M, 15	Abdominal pain & distention	Sigmoid mass (4 × 3 × 2 cm), partial obstruction	Resection of the mass and colo-colic anastomosis	Mucinous adenocarcinoma	FOLFOX protocol (Stage 3 C)/Radiotherapy	Died 14 months after the first operation
3	M, 11	Abdominal discomfort and blood in stool	Rectosigmoid mass (7.5 × 5 × 4 cm)	Total colectomy and ileo-anal anastomosis	Signet ring cell adenocarcinoma	FOLFOX protocol (Stage 3 C)/Radiotherapy	Died 4 months after the first operation
4	F, 15	Abdominal discomfort	Sigmoid mass	Resection of the mass and colo-colic anastomosis (external center)	Moderately differentiated adenocarcinoma	FOLFOX protocol (Stage 3)/Radiotherapy	Surviving for 10 years postoperatively
5	M, 15	Intermittent abdominal pain, diarrhea and blood in stool (3 months)	Sigmoid mass (10 × 4 × 3 cm)	Resection of the mass and colo-colic anastomosis	Well-differentiated adenocarcinoma	FOLFOX protocol (Stage 3)/Radiotherapy	Surviving for 4 years postoperatively

## Discussion

Pediatric CRC differs significantly from adult CRC in clinical symptoms, localization, staging, histopathologic findings, and prognosis. While factors like age, alcohol use, smoking, obesity, hypercaloric diet, red meat consumption, and sedentary living are associated with adult CRC, they are less relevant in pediatric cases. Predisposing factors also include polyposis syndromes, inflammatory bowel diseases (IBD), such as ulcerative colitis and Crohn's disease, and chemical or radiation exposure.

Most pediatric CRC cases are sporadic (65–90%), while a smaller percentage have a genetic basis [1, 5, 10–17]. The genetic causes can be classified into different types of polyposis, such as familial adenomatous polyp (FAP), MUTYH-associated polyposis, Peutz–Jeghers syndrome, juvenile polyposis syndrome, and juvenile hyperplastic polyposis syndrome. CRC can also occur as a result of the malignant transformation of IBD, specifically ulcerative colitis and Crohn's disease. Finally, Another genetic causative group is listed under hereditary non-polyposis CRC, which includes LS and the constitutional mismatch repair deficiency syndromes. Within our case series, a single case demonstrated signs of LS, whereas the remaining cases showed no indication of genetic, molecular, polyposis syndrome, or inflammatory bowel disease association.

Three mechanisms can generally explain CRC development [5, 18–20]. The first mechanism is chromosomal instability, which accounts for about 65–70% of sporadic cancers and cases of FAP. This mechanism involves the mutation of the tumor suppressor gene known as APC, leading to its inactivation. The dysregulation of e-cadherin homeostasis by the tumor suppressor gene inactivator (p53) and proto-oncogene activators (c-Myc and KRAS) initiates this mutation. The second mechanism is MSI, typically seen in LS. MSI occurs due to a mutation in the genetic line of mismatch repair (MMR) genes, with high-frequency microsatellite instability (MSI-H) being particularly significant. The third mechanism is the CpG island methylator phenotype (CIMP), which involves the hypermethylation and deletion of tumor suppressor genes like Human MLH-1 and MGMT. Abnormal methylation of MMR and MLH-1 leads to mutations in the BRAF gene at the transcription site, particularly in MSI-H cases. CIMP is observed in around 15% of sporadic CRC cases. Only one of our patients, who exhibited KRAS positivity, had MSI and demonstrated a favorable prognosis. He is in his fourth postoperative year, and his follow-up proceeds without any complications through annual laboratory, endoscopic, and radiologic examinations.

Symptoms observed in adults typically encompass changes in bowel habits, blood in stool, rectal bleeding, anemia, and weight loss. The presentation of these symptoms may differ depending on the location of the mass. In pediatric patients, the symptoms are less specific and may encompass abdominal pain, nausea, and vomiting, which can also serve as indicators for conditions, such as gastroenteritis, appendicitis, and intussusception. Advanced cases may exhibit acute abdominal conditions such as ileus or perforation. This phenomenon is more commonly observed in pediatric patients than adults, with a prevalence rate surpassing 20%. Among adults, the mass is predominantly situated in the left colon, particularly in the sigmoid and rectum regions. Children display a more equitable distribution of the mass [1, 11, 17]. In our series, three patients (60%) exhibited nonspecific symptoms, such as abdominal pain, fatigue, and nausea. Hospitalization with a diagnosis of ileus was necessary for the remaining two patients, representing 40% of the study population. Without exception, the mass was identified in the rectosigmoid colon in all our cases.

The World Health Organization (WHO) has delineated six subgroups of colorectal adenocarcinomas, which are as follows: cribriform-comedo, medullary, micropapillary, mucinous, serrated, and signet ring cell [13]. The grades of differentiation are classified into four categories: well-differentiated (Grade I), moderately differentiated (Grade II), poorly differentiated (Grade III), and undifferentiated/anaplastic (Grade IV).

Two cases exhibited mucinous characteristics in our series, and one displayed signet ring cell features. When the literature regarding CRC histology is reviewed, pediatric CRC is frequently mucinous (50% and over) and signet ring cell (48%). In contrast, adult cases have been reported to have less commonly mucinous (5–8%) and signet ring cell (1%) tumors histologically. When differentiation is considered, 32% of pediatric CRC is poorly differentiated, with a poor prognosis. Regarding differentiation grade, the ratio of poorly differentiated adult CRC cases is 18% [1, 4, 14, 18, 21]. In our series, one exhibited well-differentiated characteristics, and one exhibited moderately differentiated characteristics.

The initial investigations for the diagnosis of CRC have been recommended to include abdominal US, esophago-gastro-duodenoscopy, and colonoscopy, particularly in cases with predisposing genetic syndromes. Post-diagnosis staging investigations have been suggested to include computed thorax, abdomen, and pelvis tomographies, accompanied by Tc99 m bone scanning. Direct abdominal radiography, enema colon radiography, followed by PET, and MRI examinations provide valuable data for children. Tumor markers, such as CEA and CA 19–9, should be checked for monitoring treatment, particularly metastasis [5, 11, 22]. All our cases underwent direct abdominal radiography, US, CT, and MRI. Two patients underwent colonoscopy. Tumor markers were evaluated in all patients preoperatively, with only one exhibiting slightly elevated CA 19.9 and CA 125 levels. Postoperative follow-up of all three cases with recurrence, metastases, and poor prognosis revealed elevated CEA and CA 19–9 levels.

The most frequently employed approaches are the TNM system and Dukes'prognostic staging systems, which are recommended guidelines for CRC staging according to the American Joint Committee on Cancer (AJCC, 7 th edition) [5, 23]. Given the high frequency of regional or distant metastasis observed in children, the staging of CRC is defined as stage ≥ 3 in 86% based on the TNM system and as stages C and D in 62–86% according to Dukes'system [1, 4, 11, 14, 15]. Peritoneum (34%), liver (32%), lung (9%), ovaries (7%), and bones (7%) are the primary locations of metastasis in pediatric patients, whereas in adults, liver (30–70%), lung (20–40%), and bones (5–10%) are predominantly metastasized [1, 15].

From a histopathological perspective, mucinous or signet ring cell appearance, Stages 3 and 4 (Dukes C/D), and regional or distant metastasis indicate an unfavorable prognosis in pediatric patients. Likewise, factors including incomplete resection, presence of positive lymph nodes, and invasion of the serosa are also associated with an unfavorable prognosis. Pediatric patients'five-year survival rate varies between 23% and 65.6% [3, 5, 10, 12, 14], whereas the ten-year survival rate stands at 31% [1, 4, 5, 12]. In the adult population, the survival rate over five years ranges from 60 to 75% [3, 10, 12], whereas over ten years it is 54% [10, 12]. Based on the TNM system, all our patients were categorized as Stage 3. Nevertheless, three patients (representing 60% of the sample) displayed an unfavorable prognosis, with all three experiencing early recurrence. A combination of therapeutic modalities and multiple surgical procedures were utilized to manage diffuse metastases in the peritoneal, hepatic, and intra-abdominal regions. All three patients succumbed during the early postoperative period. Two cases of patients with a favorable prognosis (40%) have been observed, one in the fourth postoperative year and another in the tenth postoperative year, surviving without complications.

As per the literature, the treatment principles for children are recommended to align with those employed for adults. Consultation with medical oncologists with extensive experience treating adults is advised [3, 11, 13, 16, 24]. The consensus is strong regarding the pivotal role of surgery in CRC treatment, emphasizing the need for a radical surgical procedure. It is necessary to perform an extensive resection of the colon segment that harbors the tumor and its associated mesentery and draining lymphatics. Additionally, it is crucial to thoroughly explore the peritoneal surface, renal fascia, and diaphragm and to excise all peritoneal lymph nodes. Evidence in the literature supports the proficient execution of laparoscopic surgery [25].

It is advised to perform the excision of the ovary and omentum, as these areas are prone to metastatic spread. In cases of infiltration, it is recommended to perform a hysterectomy and excision of the upper vagina [11, 13, 16, 26]. It is recommended to resect at least 5 cm of normal bowel tissue on both sides to prevent potential recurrence and excise at least 12 lymph nodes to confirm the N0 stage [5, 24]. When the mass effect causes obstruction, carrying out a colostomy before radical surgery is paramount, and this approach offers a notable level of convenience when employed [13]. Resection of the mass was conducted in all our cases, including one that underwent intervention at an external center. The resection procedure involved the removal of a minimum of 5 cm of healthy bowel from both sides of the intestinal segment where the mass was situated. Additionally, all cases underwent omentectomy, appendectomy, and lymph node dissection.

Reports indicate that a considerable portion, approximately 17%, of pediatric cases cannot be surgically removed [17]. Thus, neoadjuvant radio-chemotherapy [13] or a combination of immunotherapy and chemotherapy [6] is being explored as promising treatments followed by radical surgery.

The need to tailor treatment to each patient's specific needs is emphasized. Moreover, the scarcity of experience in pediatric chemotherapy has led to using adult chemotherapy protocols [3, 5, 13, 24]. Given the predominantly advanced stage and grim prognosis of CRC in children, it is necessary to provide adjuvant chemotherapy after surgery [5]. The standard chemotherapy treatment involves the administration of 5-fluorouracil and folinic acid. The alternative agents used have been reported as capecitabine, oxaliplatin, and irinotecan [13, 24]. The effectiveness of recently developed targeted therapies based on molecular profiling has been demonstrated in several advanced cases. Bevacizumab, pembrolizumab, cetuximab, panitumumab, bortezomib, gefitinib, and cabozantinib are commonly employed agents [24, 25, 27–33]. The administration of the Folfox protocol, which included 5-fluorouracil, folinic acid, and oxaliplatin as chemotherapeutic agents [34], was implemented in all cases. Additionally, three patients with an unfavorable prognosis were augmented with methotrexate, capecitabine, and bevacizumab.

Another essential element of CRC treatment is post-surgery radiotherapy even though its applicability is limited. However, it may be deemed advisable as a preventative measure against potential reoccurrence. In addition, it has been found to have applicability before surgery in advanced-stage situations that entail obstruction or intestinal perforation [13, 26]. Radiotherapy was administered postoperatively to all the patients.

The three patients who expired during the early phases of the disease experienced several surgical procedures due to disease recurrence and ileus resulting from intra-abdominal dissemination. There is a viewpoint suggesting that hyperthermic intraperitoneal chemotherapy (HIPEC), widely utilized in successful adult surgical procedures, has the potential to be used in pediatric cases. The recent review conducted by David J. Byrwa et al. [35] has reported that the impact of HIPEC on overall survival, compared to systemic chemotherapy and mass reduction surgery, is still uncertain. This uncertainty arises from the absence of clinical trials, limited sample sizes within tumor subgroups, and the general pediatric population. Hence, elucidating the connection between the extent of tumor burden and the magnitude of surgical mass reduction is paramount. Future directions in this field encompass prospective clinical trials, establishing patient databases for standardized HIPEC in pediatric patients, and optimizing HIPEC.

## Conclusion

CRC is infrequently observed in children and is typically characterized by a poor prognosis and a late-stage diagnosis. In addition, it presents a distinct biologic structure that differs from what is observed in adults. However, the existing body of evidence is insufficient for accurately predicting survival. The suboptimal results obtained by applying adult treatment protocols to children reinforce the importance of conducting new studies. Future research endeavors should focus on discovering innovative biomolecular indicators and formulating novel therapeutic approaches.

## Data Availability

The data that support the findings of this study are available from the corresponding author, upon reasonable request.

## Abbreviations

AJCC: American Joint Committee on Cancer
CA 19.9: Carbohydrate antigen 19–9
CA 125: Carbohydrate antigen 125
CEA: Carcinoembryonic antigen
CIMP: CpG island methylator phenotype
CRC: Colorectal cancer
CT: Computed tomography
FAP: Familial adenomatous polyp
Folfox protocol: 5-Fluorouracil, Folinic acid, and Oxaliplatin
HIPEC: Hyperthermic intraperitoneal chemotherapy
IBD: Inflammatory bowel diseases
LS: Lynch syndrome
MLH-1: Human mutL homolog 1
MMR: Mismatch repair
MRI: Magnetic resonance imaging
MSH-2: Human mutS homolog 2
MSH-6: Human mutS homolog 6
MSI: Microsatellite instability
MSI-H: High-frequency microsatellite instability
PET: Positron emission tomography
PMS-2: Human postmeiotic segregation increased 2
US: Ultrasound
WHO: World Health Organization
